# Cytotoxicity of synthetic derivatives against breast cancer and multi-drug resistant breast cancer cell lines: a literature-based perspective study

**DOI:** 10.1186/s12935-021-02309-9

**Published:** 2021-11-20

**Authors:** Shabnam Sharmin, Md. Mizanur Rahaman, Miquel Martorell, Jorge Sastre-Serra, Javad Sharifi-Rad, Monica Butnariu, Iulia Cristina Bagiu, Radu Vasile Bagiu, Mohammad Torequl Islam

**Affiliations:** 1 Department of Pharmacy, Life Science Faculty, Bangabandhu Sheikh Mujibur Rahman Science and Technology University, Gopalganj (Dhaka), 8100 Bangladesh; 2 Department of Nutrition and Dietetics, Faculty of Pharmacy, and Centre for Healthy Living, University of Concepción, 4070386 Concepción, Chile; 3 Grupo Multidisciplinar de Oncología Traslacional, Institut Universitari d’Investigació en Ciències de La Salut (IUNICS), Universitat de Les Illes Balears, Palma de Mallorca, Illes Balears Spain; 4 Instituto de Investigación Sanitaria de Las Islas Baleares (IdISBa), Hospital Universitario Son Espases, Edificio S, 07120 Palma de Mallorca, Illes Balears Spain; 5 Ciber Fisiopatología Obesidad y Nutrición (CB06/03), Instituto Salud Carlos III, 28029 Madrid, Spain; 6 Phytochemistry Research Center, Shahid Beheshti University of Medical Sciences, Tehran, Iran; 7 Banat’s University of Agricultural Sciences and Veterinary Medicine “King Michael I of Romania” From Timisoara, Timisoara, Romania; 8 Department of Microbiology, Victor Babes University of Medicine and Pharmacy of Timisoara, Timisoara, Romania; 9 Multidisciplinary Research Center On Antimicrobial Resistance, Timisoara, Romania; 10 Preventive Medicine Study Center, Timisoara, Romania

**Keywords:** Synthetic derivatives, Breast cancer cell line, MDR breast cancer cell line, Cytotoxicity

## Abstract

Cancer is the second most killer worldwide causing millions of people to lose their lives every year. In the case of women, breast cancer takes away the highest proportion of mortality rate than other cancers. Due to the mutation and resistance-building capacity of different breast cancer cell lines against conventional therapies, this death rate is on the verge of growth. New effective therapeutic compounds and treatment method is the best way to look out for in this critical time. For instance, new synthetic derivatives/ analogues synthesized from different compounds can be a ray of hope. Numerous synthetic compounds have been seen enhancing the apoptosis and autophagic pathway that directly exerts cytotoxicity towards different breast cancer cell lines. To cease the ever-growing resistance of multi-drug resistant cells against anti-breast cancer drugs (Doxorubicin, verapamil, tamoxifen) synthetic compounds may play a vital role by increasing effectivity, showing synergistic action. Many recent and previous studies have reported that synthetic derivatives hold potentials as an effective anti-breast cancer agent as they show great cytotoxicity towards cancer cells, thus can be used even vastly in the future in the field of breast cancer treatment. This review aims to identify the anti-breast cancer properties of several synthetic derivatives against different breast cancer and multi-drug-resistant breast cancer cell lines with their reported mechanism of action and effectivity.

## Introduction

Cancer is typically a heterogeneous disease and one of the second dominant causes of morbidity and mortality around the globe [1, 2]. This disease revolves around unnatural cell proliferation which may or may not invade the other parts of the body. Among all the cancer types, breast cancer is most deadly for women and also contributes to the highest mortality rate when compared to other types [3–7]. According to World Health Organization (WHO) breast cancer is very persistent in women, affecting about 2.3 million each year. In 2020, approximately 685,000 women died from this disease [8]. Estrogen receptor beta (ERβ) has been marked as a possible origin of developing breast cancer and around 60% of breast cancer is hormone-dependent, relying on estrogen for growth [3, 9, 10]. Abnormality and irregularity in the normal cell cycle along with obstructed apoptosis signaling pathway is the fundamental cause for breast cancer progression [11–13]. A subtype of breast cancer investigated as triple-negative breast cancer (TNBC) is a result of a shortfall of expression of estrogen receptor alpha/progesterone receptor [9, 14, 15].

**Fig. 1:**
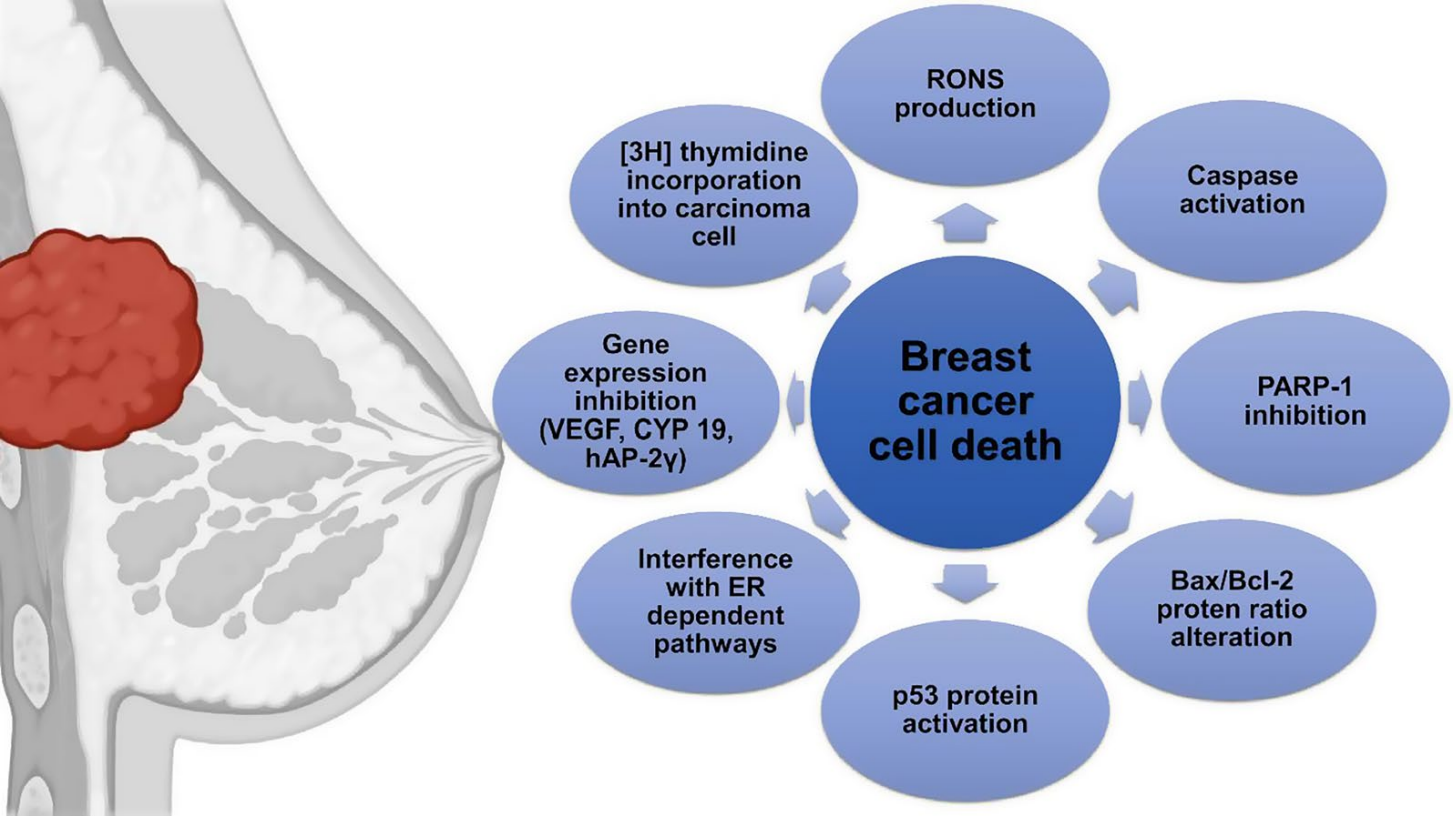
Mechanisms of breast cancer cell death. CYP cytochrome, ER endoplasmic reticulum, hAP-2γ human transcription factor activation protein-2 γ, PARP-1 poly [ADP-ribose] polymerase 1, RONS reactive oxygen and nitrogen species, VEGF vascular endothelial growth factor

As for the treatment’s concern, radiation therapy, chemotherapy, hormone therapy, and targeted therapy are often used alongside surgery for early-stage patients [16–18]. Patients with metastatic disease are also treated the same way with systemic therapy which recently included immunotherapy [18]. Most of these therapies incorporate apoptosis or programmed cell death to instigate the anti-breast cancer activity throughout development, differentiation, tumor cell detection, and in response to specific cytotoxicity of molecules or compounds [19–22]. This programmed cell death follows an intrinsic or extrinsic pathway that comes with a series of occurrences including the altered ratio of Bax/Bcl-2 protein, activated caspases, and bifurcated poly [ADP-ribose] polymerase (PARP-1) enzyme [21, 23–27]. Generation of reactive oxygen species (ROS) and formation of nitric oxide (NO) also leads to p53 activation which results in DNA damage of cancer cells [28–31]. Autophagy, a cellular homeostasis mechanism may also contribute to breast cancer cell death where autophagosomes amalgamate with the lysosome to establish autophagolysosome during starvation and stress [32]. PARP-1 enlivening and LC3-II protein marker urges autophagic cell death [33, 34]. Figure 1 summarizes the mechanisms involved in breast cancer cell death.

Considering the complexity of the disease and the paucity of an effective chemotherapeutic agent, breast cancer besides other cancers has drawn the attention of researchers. Many of these researches have pointed towards chemotherapeutic agents that have been procured from natural or synthetic origin [21]. A slight modification in the structure of the natural compound or by the synthesis of specific analogues worthwhile activities is seen in the case of cancer therapy. Paclitaxel, vinca alkaloids, camtothecin, and etoposide are some of the synthetic derivatives vastly used for cancer therapy originally attained from natural sources [35]. Synthetically derived substances for cancer therapy are highly being studied in a hope that they might tame the unexpected and unavoidable side-effects originated by chemotherapeutic drugs [36]. A Wyrębska, K Gach, U Lewandowska, K Szewczyk, E Hrabec, J Modranka, R Jakubowski, T Janecki, J Szymański and A Janecka [37] reported the anti-breast cancer activity of synthetically derived α-methylene-δ-lactones on hormone-independent MDA-MB-231, hormone-dependent MCF-7 cell lines through intrinsic apoptotic pathway activation, cancer cell migration suppression, and invasion. Synthetic vitamins, curcuminoids, isoflavones, chromenes are also seemed to have anti-breast cancer activity when tested on different cell lines [38–44].

Another vital road-blocker is the development of resistance that calls for never-ending neediness for new therapeutics [45–47]. Multi-drug resistance (MDR), the main fundamental cause behind chemotherapy failure may develop due to some complex mechanism including transporter-mediated efflux, over-expression of efflux transporters: P-glycoprotein (ABCB-1/P-gp), breast cancer resistance protein (BCRP), and multidrug resistance-associated proteins (MRPs) present on the cell membrane [48–55]. Efflux transporters effectively pump out drugs that are meant to create cytotoxicity in the cell. As a result, the intracellular concentration of that specific drug fall. MDR cancer cells containing efflux or ATP-binding cassette (ABC) transporters can significantly interact or deliver a plethora of anticancer compounds using the hydrophobic vacuum cleaner mode where the hydrophobic compounds get attach to the MDR-1 on account of their hydrophobicity for efflux [56]. In the case of a pump-independent mechanism, the cellular anti-apoptotic defense system activation develops resistance toward chemotherapeutic agents by upregulating BCL2 gene [57]. Evidence shows that synthetically derived compounds effectively exert cytotoxicity on MDR cancer cells. Zhou et al. [58], stated that synthetically derived β-amino ester inhibits P-gp activity by lowering mitochondrial membrane potentials and ATP levels on MCF-7 cell line. The enhanced antitumor effect might be attributed to PHP-mediated lysosomal escape and drug efflux inhibition.Various other studies show a similar effect on different tested cell lines.

Traditionally available chemotherapeutic agents may develop undesirable side effects and sometimes may also lack efficacy. So, new and advanced sources are in need that may counterbalance the present difficulties. In this study, the cytotoxic effect of different synthetic derivatives on normal and MDR cell lines is thoroughly discussed. This review set the sights on drawing the attention of researchers to conduct more advanced level analysis on the cytotoxicity of these synthetically derived analogues.

## Methodology

A search (till February 2021) was done in the following databases: PubMed, Science Direct, MedLine, and Google Scholar with the keyword ‘Synthetic derivative’, paring with ‘against breast cancer cell line/ multi-drug-resistant breast cancer cell lines or cytotoxicity on breast cancer/ multidrug-resistant cell line. No language restrictions were imposed. Articles were assessed for information about the synthetic derivatives, breast cancer cell lines, multi-drug-resistant breast cancer cell lines, test results, and possible mechanisms of action.

### Inclusion criteria

The following inclusion criteria were adopted:

1. Studies with synthetic derivatives/analogues from various sources.
2. Studies carried out in vivo, in vitro, or ex vivo on breast cancer cells/ multi-drug-resistant breast cancer cells.
3. Studies with or without activity mechanism.

### Exclusion criteria

The following exclusion criteria were adopted:

1. Titles and/or abstract not meeting the inclusion criteria, duplication of data.
2. Synthetic derivatives with other studies obscuring the current subject of interest.

## Findings

Among the vast pieces of evidence, some randomly selected published articles found in the databases that contain screening reports on synthetic derivatives acting against breast cancer/ MDR cell-line have been summarized below:

### Cytotoxicity of synthetic derivatives on different breast cancer cell lines

Synthetic derivatives in a similar manner tonatural substances follow apoptosis and autophagic pathways to inhibit the growth and activity of breast cancer cells. Other than that inhibition of cell proliferation, induction of cell-cycle arrest may occur. AM Oliveira Rocha, F Severo Sabedra Sousa, V Mascarenhas Borba, SM T, J Guerin Leal, OE Dorneles Rodrigues, GF M, L Savegnago, T Collares and F Kömmling Seixas [59] reported the anti-breast cancer activity of synthetic azidothymidine (AZT) derivatives containing tellurium (Te) on MDA-MB-231 cell-line using MTT assay. The derived compounds 7 m and 7r showed an inhibitory effect on the breast cancer cell-line through lowering cell proliferation, initiating cell-cycle arrest in the S phase in the absence of the apoptosis process. Subsequently, the synthetic drug pair, piperidinyl-diethylstilbestrol (DES), pyrrolidinyl-DES exhibits cytotoxicity on MCF-7 cell-line in both in vivo and in vitro assay. In the case of the in vitro study, these drugs manifest cytotoxicity on shrimp larvae at LC_50_ 19.7 ± 0.95 and 17.6 ± 0.4 μg/mL respectively. In vivo cell inhibition is seen by ceasing G0/G1-phase of the MCF-7 cell cycle following ED_50_ value 7.9 ± 0.38 and 15.6 ± 1.3 μg/mL [36].

The induction of apoptotic pathways can be an effective course of action to inhibit cancer cells. Studies reported a heap of incidences where apoptosis effectively took part in breast cancer cell destruction [38, 60, 61]. Kheirollahi et al. [39] reported the anti-breast activity of synthetic benzochromene derivatives on 3 different breast cancer cells (MCF-7, MDA-MB-231, and T-47D) where the derivatives participate in ROS and NO production through direct modification of proteins, lipids, and DNA that induces apoptosis in cancer cell lines. To that add this, synthetic oleanolic acid derivative HIMOXOL induced apoptotic pathway by activating caspase-8, caspase-3, and PARP-1 protein, elevating the ratio of Bax/Bcl-2 protein level, triggering microtubule-associated protein LC3-II expression, and upregulating bectin 1 on MDA-MB-231 cell-line at IC_50_ value 7.33 ± 0.79 μM [62].

Autophagic pathway activation by synthetic derivatives is also marked as a potential solution in the case of cancer cell inhibition. Synthetic β-nitrostyrene derivative, CYT-Rx20 shows inhibitory activity on MCF-7, MDA-MB-231, and ZR75-1 cell-line with IC_50_ value 0.81 ± 0.04, 1.82 ± 0.05, and 1.12 ± 0.06 μg/mL respectively. The cytotoxic mechanism behind this can be illustrated as arrested cancer cells at the G2/M phase, decreased cell viability by activating caspase cascade, increased PARP cleavage, and γ-H2AX expression as well as induced autophagy by upregulation of Bectin-1, autophagy related 5 (ATG5), LC-3, and formation of ROS [63].

[3H] Thymidine is often incorporated into the daughter strands of DNA during the mitotic cell division process. As [3H] thymidine may directly calculate the proliferation so inhibition of incorporation often points towards anti-proliferative activity [64]. Synthetic derivatives effectively inhibit [3H] thymidine incorporation into the breast cancer cell to promote activity. Wyrębska et al. [65] stated that synthetic derivative MZ-6 inhibited incorporation of [3H] thymidine dose-dependently alongside induced apoptosis into MCF-7, MDA-MB-231 breast cancer cell line. Furthermore, Synthetic caffeic acid phenethyl ester (CAPE) isolated from propolis shows a similar result when tested upon MCF-7 at IC_50_ 5 μg/mL [66].

**Fig. 2:**
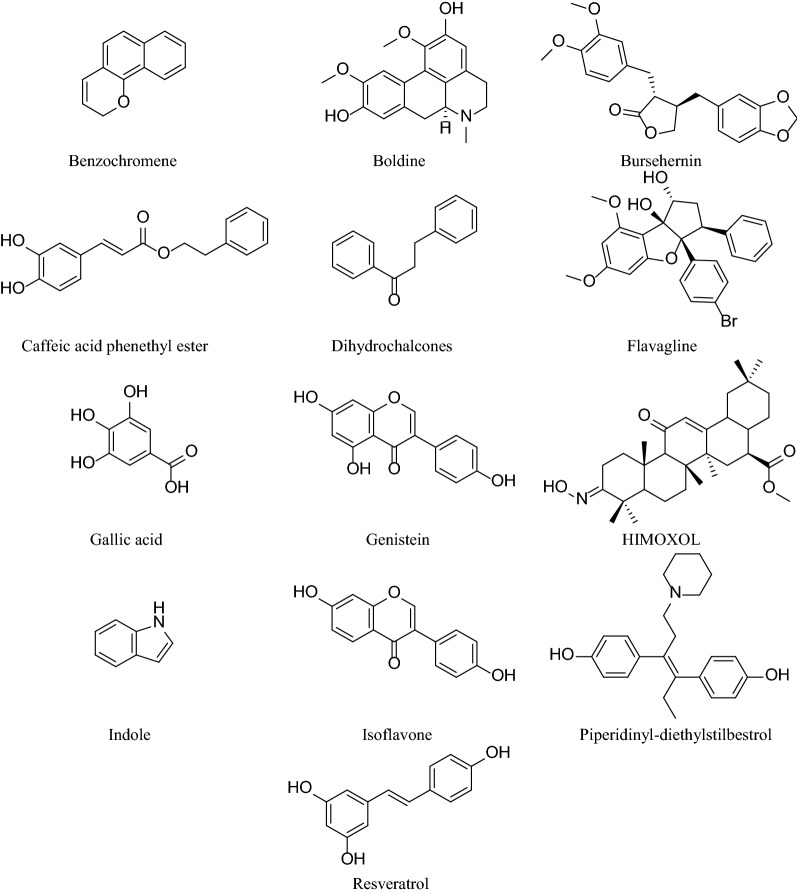
Chemical structure of some synthetic derivatives that acting against different breast cancer cell lines

Table 1 summarizes the synthetic derivatives acting against different breast cancer cell lines and Fig. 2 represents the chemical structures of these compounds.

**Table 1:** Synthetic derivatives acting against different breast cancer cell lines

Synthetic derivatives	Breast cancer cell-line	Inhibitory concentration (IC_50_)/ Lethal concentration (LC_50_)	Mechanism of action	References
Synthetic azidothymidine (AZT) derivatives containing tellurium (Te)	MDA-MB-231	**7 m:** 24.95 ± 6.05 µM (24 h), 11.76 ± 2.97 µM (48 h) **7r:** 21.61 ± 2.44 µM (24 h), 9.62 ± 1.35 µM (48 h)	Decreased cell proliferation rate, and promotion of cell cycle arrest in the S phase	[59]
Synthetic α-Methylene-δ-Lactones	Hormone-independent MDA-MB-231, hormone-dependent MCF-7	**DL-1:** 11.4 ± 2.10 µM (MDA-MB-231), 8.17 ± 0.58 µM (MCF-7) **DL-2:** 15.1 ± 1.82 µM (MDA-MB-231), 12.67 ± 0.29 µM (MCF-7) **DL-3:** 5.3 ± 0.69 µM (MDA-MB-231), 3.54 ± 0.76 µM (MCF-7) **DL-4:** 7.9 ± 0.99 µM (MDA-MB-231), 4.75 ± 1.09 µM (MCF-7)	The activated intrinsic pathway of apoptosis by loss of mitochondrial membrane potential, and change in Bax/Bcl-2 ratio, the inhibited movement of both types of cancer cells, suppressed cell migration and invasion due to decreased secretion of enzymes that cause degradation of cellular matrix, MMP-9, and uPA	[37]
Piperidinyl-diethylstilbestrol, Pyrrolidinyl-diethylstilbestrol	MCF-7	**Piperidinyl diethylstilbestrol:** 19.7 ± 0.95 μg/mL (LC50, in vitro), 7.9 ± 0.38 μg/mL (ED50, in vivo) **Pyrrolidinyl diethylstilbestrol:** 17.6 ± 0.4 μg/mL (LC50, in vitro), 15.6 ± 1.3 μg/mL (ED50, in vivo)	Exhibited toxicity and cytotoxicity of synthetic compounds on shrimp larvae, and cell culture, inhibited G0/G1-phase of the MCF-7 cell cycle	[36]
A synthetic curcuminoid, (Z)-3-hydroxy-1-(2-hydroxyphenyl)-3-phenylprop-2-en-1-one (DK1)	MCF-7 and compared with MDA-MB-231 and MCF-10	**24 h:** 96.83 ± 4.87 µM (MCF-7), 104.17 ± 5.23 µM (MDA-MB-231), > 208 µM (MCF-10) **48 h:** 33.33 ± 3.50 µM for MCF-7, 45.83 ± 4.66 µM (MDA-MB-231), 125.38 ± 3.67 µM (MCF-10) **72 h:** 25 ± 3.71 µM (MCF-7), 37.50 ± 4.82 µM (MDA-MB-231), 104.17 ± 5.21 µM (MCF-10)	Induced cytotoxicity against MCF-7 breast cancer cells, induced p53 mediated apoptosis through ROS induction, and inhibition of GSH, induced G2/M cell cycle arrest through up-regulating p21, and down-regulating PLK-1	[38]
Synthetic antiestrogen 4-hydroxytamoxifen (OH-Tam), antiprogestin 17β-hydroxy-11β-(4-methylaminophenyl)-17-(1-propynyl)estra-4,9-dien-3-one-6–7 (RU486)	MCF-7, MDA-MB-231, BT20		A triggered third type of receptor-mediated cytotoxicity by antiestrogens. Similar activity was seen for antiprogestin indicating anti-hormone, and antiproliferative effect	[67]
Synthetic Vit-E supplement, dl-α-tocopherol	MDA-MB-231	Not mentioned	Reduced lipid peroxidation results in suppressed tumor growth. Stabilized membrane fatty acids in the acyl chain show antitumor activity	[40]
Synthetic isoflavones (**1**, **2**, **3**, **4**, **5**, **6**, **7**, **8**, **9**, **10**)	Hormone-independent MDA-MB-231, hormone-dependent MCF-7	**1:** 11.1 ± 5.0 µM **2:** 8.2 ± 2.0 µM **5:** 0.04 ± 0.01 µM **6:** 6.3 ± 1.0 µM **7:** 2.1 ± 0.4 µM **9:** 1.8 ± 0.6 µM **10:** 2.9 ± 0.2 µM	Activated mechanism of celldeath and affected breast cancer cell survival by acting on multiple signaling pathways	[41]
Synthetic caffeic acid phenethyl ester (CAPE) isolated from propolis	MCF-7	Incorporation of [3H] thymidine into the DNA of human breast carcinoma MCF-7 is 50% inhibited at 5 μg/mL CAPE	Inhibited incorporation of [3H] thymidine into carcinoma cell results in cytotoxic activity	[66]
Synthetic derivatives of benzochromene, **4a**, **4b**, **4c**, **4d**, **4e**	MCF-7, MDA-MB-231, T-47D	**4a:** 9.9 ± 0.57 μM (MCF-7), 11.7 ± 1.8 μM (MDA-MB-231), 6.9 ± 0.65 μM (T-47D) **4b:** 10.3 ± 0.58 μM (MCF-7), 6.1 ± 2.3 μM (MDA-MB-231), 5.3 ± 0.66 μM (T-47D) **4c:** 9.3 ± 0.61 μM (MCF-7), 6 ± 0.7 μM (MDA-MB-231), 8.7 ± 0.55 μM (T-47D) **4d:** 11.07 ± 0.87 μM (MCF-7), 18.1 ± 1.8 μM (MDA-MB-231), 6.9 ± 0.67 μM (T-47D) **4e:** 11.6 ± 0.44 μM (MCF-7), 21.5 ± 1.8 μM (MDA-MB-231), 4.6 ± 0.068 μM (T-47D)	Increased ROS and NO production through direct modification of proteins, lipids, and DNA that induces apoptosis in cancer cell lines	[39]
Synthetic oleanolic acid derivative, Methyl 3-hydroxyimino-11-oxoolean-12-en-28-oate (HIMOXOL)	MDA-MB-231	**24 h:** 21.08 ± 0.24 μM **72 h:** 7.33 ± 0.79 μM	Increased apoptotic pathway via activation of caspase-8, caspase-3, and PARP-1 protein, increased ratio of Bax/Bcl-2 protein level, triggered microtubule-associated protein LC3-II expression, and upregulated bectin 1	[62]
Four groups of synthetic derivatives of isoliquiritigenin analogues including, hydroxy-substituted chalcones (2a-2f), chalcones substituted with methoxy group (3a-3 l), flavanones (4a-4b), dihydro-chalcones (5a-5c)	MCF-7, MDA-MB-231	IC50 < 10 μM are shown **3c:** 1.5 ± 0.18 μM (MCF-7), 7.9 ± 1.0 μM (MDA-MB-231) **3d:** 3.1 ± 0.65 μM (MCF-7), > 10 μM (MDA-MB-231) **3f:** > 10 μM (MCF-7), 6.6 ± 0.75 μM (MDA-MB-231) **3 g:** > 10 μM (MCF-7), 7.4 ± 1.16 μM (MDA-MB-231) **3 h:** 0.71 ± 0.17 μM (MCF-7), 6.5 ± 0.83 μM (MDA-MB-231) **3 l:** 7.0 ± 1.54 μM (MCF-7), > 10 μM (MDA-MB-231)	The second group showed antitumor activity. Methylated hydroxyl groups in chalcones escalated the cytotoxic activity	[68]
Synthetic genistein glycosides, G15, G16, G17, G21, G23, G24, G26, G30, G31	MCF-7	LC50 values: G15: 34 μM G21: 45 μM G23: 32 μM G24: 43 μM G26: 63 μM G30: 51 μM G31: 67 μM	Increased lipophilicity, acetylated sugar hydroxyls, directedly bound double CC bond in sugar to aglycone, α configured genistein-sugar glycoside bond, localized sugar substituent at the 7-OH position in genistein molecule contributes to the cytostatic/ cytotoxic activity	[69]
Synthetic conjugates of genistein, Ram-3 (8b)	MCF-7, SKBR-3	**Ram-3:** 8.88 ± 0.75 μM (MCF-7) 28.02 ± 6.89 μM (SKBR-3)	Inhibited cellcycle, interaction with mitotic spindles, and apoptotic cell death leads to cancer cell anti-proliferative activity	[70]
Synthetic flavagline, 3 (FL3)	MCF-7	**FL3:** 1 μM	Induced cancer cell death via activation of the apoptosis-inducing factor and caspase-12 pathway	[71]
Synthetic peptides derived from Bovine lactoferricin sequences, LfcinB (20–25): 20RRWQWR25, LfcinB (20–30): 20RRWQWRMKKLG30, and [Ala19]-LfcinB (17–31): 17FKARRWQWRMKKLGA31 containing (i) a linear; (ii) a dimeric; (iii) a cyclic; (iv) a tetrameric peptide	MDA-MB-468, MDA-Mb-231	Only tetrameric and dimeric peptides showed cytotoxicity against both cancer cells **LfcinB (20–25)4:** 6 μM (MDA-MB-468), 15 μM (MDA-Mb-231) **LfcinB (20–30)2:** 5 μM (MDA-MB-468), 14 μM (MDA-Mb-231) **LfcinB (20–30)4:** 2 μM (MDA-MB-468), 6 μM (MDA-Mb-231) **[Ala19]-LfcinB (17–31)2:** 11 μM (MDA-MB-468), 31 μM (MDA-Mb-231) **[Ala19]-LfcinB (17–31)4:** 5 μM (MDA-MB-468), 9 μM (MDA-Mb-231)	Not mentioned	[72]
Synthetic 3-isopropyl-2-methyl-4-methyleneisoxazolidin-5-one (MZ-6)	MCF-7, MDA-MB-231	**MZ-6:** 7.25 μM (MCF-7) 6.5 μM (MDA-MB-231)	Inhibited incorporation of [3H]thymidine dose-dependently, up-regulated Bax, and down-regulated Bcl-2 mRNA, elevated end products of lipid peroxidation, malondialdehyde results in apoptosis and cell-cycle arrest in G0/G1 phase	[65]
Synthetic diterpene 1, 2	MCF-7, NCI/ADR/RES, MDA-MB-231, HS 578 T, MDA-MB-435, BT-549, T-47D	**1:** > 100 μMC50 for all cell-lines **2:** 26.6 μM (MCF-7) 28.3 μM (NCI/ADR/RES) 34.6 μM (MDA-MB-231) > 50.0 μM (HS 578 T) 37.7 μM (MDA-MB-435) > 50.0 μM (BT-549) 39.7 μM (T-47D)	Inhibited cancer cell proliferation results in cytostatic activity	[73]
Synthetic derivatives of novel N-substituted bis-benzimidazole, 9a, 9b, 9c, 9d, 9e, 9f, 9 g, 9 h, 9i	MCF-7, MDA-MB-453	**9c:** 52.09 µg/mL(MCF-7), 55.89 µg/mL (MDA-MB-453) **9 g:** > 100 µg/mL (MCF-7), > 100 µg/mL (MDA-MB-453) **9i:** > 100 µg/mL (MCF-7), > 100 µg/mL (MDA-MB-453)	Well-documented apoptosis or programmed cell death is the key mechanism to exert cytotoxicity	[74]
Synthetic ( ±)-kusunokinin and its derivative ( ±)-bursehernin	MCF-7, MDA-MB-468, MDA-MB-231	**( ±)-kusunokinin:** 4.30 ± 0.65 μM (MCF-7), 5.90 ± 0.44 μM (MDA-MB-468), 7.57 ± 0.92 μM (MDA-MB-231) **( ±)-bursehernin:** 11.96 ± 0.62 μM (MCF-7), 8.24 ± 0.08 μM (MDA-MB-468), 14.26 ± 0.61 μM (MDA-MB-231)	Suppressed STAT3 and topoisomerase II including cell-cycle arrest and apoptosis through multi-caspase activity including caspase-1, -3, -4, -5, -6, -7, -8, and -9	[75]
Synthetic ginsenoside-M1 (5) and synthetic three novel mono-esters ginsenoside-DM1 (6), PM1 (7), and SM1 (8)	MCF-7	**M1 (5):** 8.48 μg/mL **DM1 (6):** 0.50 μg/mL **PM1 (7):** 2.31 μg/mL **SM1 (8):** 1.65 μg/mL	Inhibited cell proliferation and induced apoptosis lead to cytotoxic activity	[76]
A synthetic derivative of ursolic acid, FZU3010	SUM149PT, HCC1937	4–6 μM	Induced cell-cycle arrest at S and G0/G1 phase show apoptotic activity	[77]
Synthetic derivatives of novel ursolic acid containing an acyl piperazine moiety, 4b, 4c, 4d, and 4 k	Bcap-37	**4b:** 9.24 ± 0.53 μM **4c:** 4.32 ± 0.42 μM **4d:** 7.26 ± 0.46 μM **4 k:** 5.34 ± 0.41 μM	Incorporated acyl piperazine moiety at C-28 while maintaining the polar group at C-3 effectively improves the antitumor activity of the compounds	[78]
Synthetic derivatives of hexahydrobenzo [g]chromen-4-one, (7a-7 k)	MCF-7, MDA-MB-231, T-47D	Lowest values for each cell-line are shown below: **(MCF-7):** **7e:** 3.1 ± 0.8 μg/mL **7 g:** 3.3 ± 0.1 μg/mL **(MDA-MB-231):** **7 h:** 2.4 ± 0.6 μg/mL **7e:** 2.5 ± 0.8 μg/mL **(T-47D):** **7 h:** 1.8 ± 0.6 μg/mL **7 g**: 2.9 ± 0.9 μg/mL	Induced apoptosis, increased ROS, and NO production	[42]
Synthetic derivatives of 2-aryl-3-nitro-2H-chromene, (4a-4u)	MCF-7, T-47D, MDA-MB-231	**MCF-7:** **4 l:** 0.2 ± 0 μM **4 h:** 1.6 ± 0.2 μM **T-47D:** **4c:** 2.1 ± 0.9 μM **MDA-MB-231:** **4b:** 0.4 ± 0.2 μM **4 m:** 0.5 ± 0.2 μM	Induced apoptosis by the unsubstituted and 8-methoxylated chromene series	[43]
Synthetic derivatives of boldine, (2–4)	MCF-7, MDA-MB-231	**2:** > 100 μM for both cell-lines **3:** 96.4 ± 14.2 μM (MCF-7), 100.2 ± 9.5 μM (MDA-MB-231) **4:** 64.8 ± 4.2 μM (MCF-7), 70.2 ± 5.7 μM (MDA-MB-231)	Inhibited cancer cell growth	[79]
Synthetic gallic acid-based indole derivatives, (2a, 3a, 3b, 3c, 3d, 3e, 3f, 7a)	MCF-7	**3e:** 19.2 ± 1.1 μM **3f:** 13.3 ± 0.9 μM	Observed a limited degree of agreement between cytotoxic and antioxidant activity. Position of imine link and different substituents on indole moiety contributes to the cell cytotoxicity	[80]
Synthetic steroid derivatives, (8, 12, 17, 20, 22c, 24c, 30a, and 30b)	MCF-7	**8:** 7.5 μM **17:** 2.5 μM **20:** 4.7 μM **22c:** 7.3 μM Result for 48 h incubation	Decreased breast cancer-related geneexpression (VEGF, CYP19, and hAP-2γ)	[81]
Synthetic β-nitrostyrene derivative, CYT-Rx20	MCF-7, MDA-MB-231, ZR75-1	**CYt-Rx20:** 0.81 ± 0.04 μg/mL (MCF-7) 1.82 ± 0.05 μg/mL (MDA-MB-231) 1.12 ± 0.06 μg/mL (ZR75-1)	Arrested cancer cells at the G2/M phase, decreased cell viability by activating caspase cascade, increased PARP cleavage, and γ-H2AX expression, induced autophagy by upregulation of Bectin-1, ATG5, LC-3, and formation of ROS results in cell death	[63]
Synthetic derivatives of thiazolidin-based resveratrol, (3–14)	MCF-7, SKBR-3	**9:** 2.58 μM (MCF-7) **10:** 5 μM (MCF-7) **12:** 0.81 μM (SKBR-3) **13:** 0.25 μM (SKBR-3) **14:** 0.23 μM (SKBR-3)	Interfered ER**α** -dependent pathway of ER-positive MCF-7 cells by 9–10 compounds and antagonized GPER-dependent pathway of ER-negative and GPER positive SKBR-3 cells by 12–14 compounds (under investigation)	[82]
Synthetic derivatives of (1,3)dioxolo[4,5-g]chromen-8-one, (4a–4e)	MCF-7, T-47D, MDA-MB-231	**4a:** 6.2 ± 0.1 μg/mL (MCF-7) 4.6 ± 0.1 μg/mL (T-47D) 9.3 ± 2.1 μg/mL (MDA-MB-231) **4b:** 5.7 ± 0.007 μg/mL (T-47D)	Induced apoptosis in the cancer cell lines	[44]

*ATG5* Autophagy related 5, *CYP* cytochrome, *ER* estrogen receptor, *GPER* G protein-coupled estrogen receptor, *hAP-2γ* human transcription factor activation protein-2 γ, *H2AX* H2A histone family member X, *MMP-9* matrix metallopeptidase 9, *NO* nitric oxide, *PARP* poly [ADP-ribose] polymerase, *PLK-1* polo-like kinase, *ROS* reactive oxygen species, *STAT3* signal transducer and activator of transcription 3, *uPA* urokinase plasminogen activator, *VEGF* vascular endothelial growth factor

### Cytotoxicity of synthetic derivatives on different multi-drug resistant (MDR) cancer cell lines

**Fig. 3:**
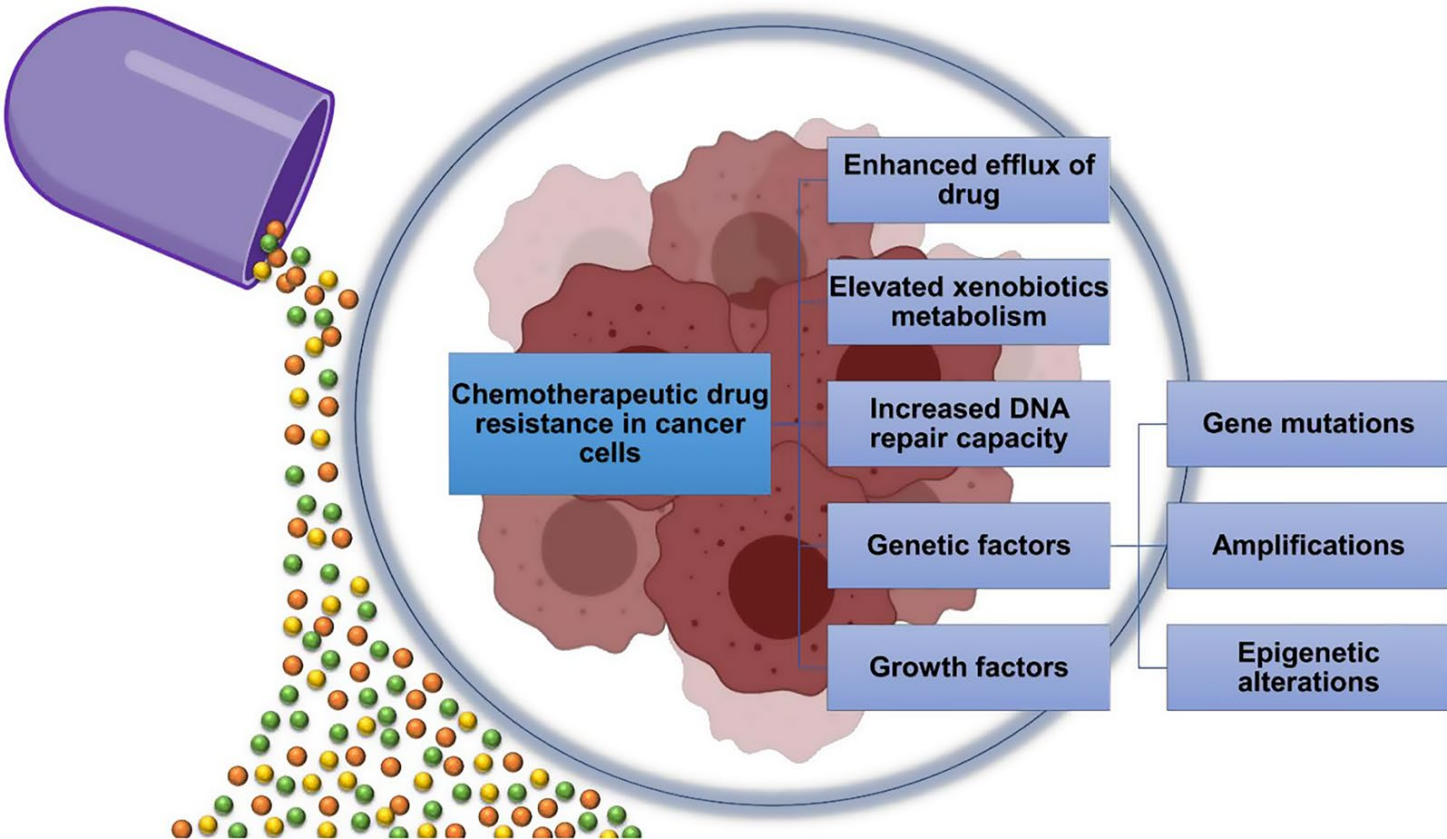
Mechanisms of chemotherapeutic drug resistance in cancer cells

Resistance against drugs used for a specific purpose can be a hugely troublesome matter when it comes to the treatment of a serious disease like cancer. Not only in the case of treatment but also in the case of the development of new therapeutics, “Multi-drug resistance” can be an invisible obstacle in pharmacology [83]. The resistance of tumor cells towards chemotherapeutic agents, leading to the failure of cancer treatment can be defined as MDR [45, 46]. MDR of cancer cells during chemotherapy should be associated with a different type of mechanisms that are including enhanced efflux of drugs, genetic factors (gene mutations, amplifications, and epigenetic alterations), growth factors, increased DNA repair capacity, and also elevated metabolism of xenobiotics (Fig. 3). In the case of breast cancer, advancements in treatment and prevention have taken place over the last decade but MDR has been witnessed as the main roadblock [48]. In recent years, the use of different synthetically derived substances has been seen effective against MDR breast cancer cells.

One of the major reasons for MDR is the over-expression of P-gp, a protein encoded by the MDR-1 gene belonging to the ABC membrane transporters family. HB Xu, L Li and GQ Liu [84] reported that a synthetic derivative Guggulsterone shows an MDR-reversal effect, a valuable adjunct to chemotherapy. Increased intracellular accumulation of Doxorubicin, an anti-breast cancer drug, results in the expression Guggulsterone in both MRP1 and P-gp in drug-resistant MCF-7 cells. Again sphingosine stereoisomers, another synthetic compound reduces basal phosphorylation of the P-gp ion in MCF-7/ADR cells, suggesting inhibition of protein kinase C (PKC)-mediated phosphorylation of P-gp [85]. 1,4-Dihydropyridines (DHPs) 3-pyridyl methyl carboxylate and alkyl carboxylate moieties inhibited rhodamine 123 efflux showing the mechanism of MDR reversal in P-gp transporter modulation. Lowered resistance of MES-SA/DX5 to doxorubicin also exerted the anti-tumor effect in MCF-7ADR cells [86].

Additionally, induction of apoptosis and autophagy can be effective ways to look out for. Genistein at IC_50_ value 73.89 µM showed an anti-tumor effect against MCF-7 cells. Induced cell-cycle arrest and apoptosis caused by genistein treatment strongly inhibits HER2/neu but not MDR-1 expression at both the mRNA and protein levels. Geinstein acts synergistically with doxorubicin by increasing intracellular accumulation of doxorubicin and suppressed HER2/neu expression [87]. M Distefano, G Scambia, C Ferlini, C Gaggini, R De Vincenzo, A Riva, E Bombardelli, I Ojima, A Fattorossi, PB Panici, et al. [88] stated that a series of14β-hydroxy-10-deacetylbaccatin III (14-OH-DAB) analogues induce cell cycle block at G2/M in a concentration-dependent manner. G1/G2 ratio, measured as the amount of cell block correlates significantly (p < 0.001) with apoptosis, evaluated in the sub-G1 region. This incident suggests G2/M-blocked cells underwent apoptosis in both MDA-MBA-231, MCF-7ADRr cells.

**Fig. 4:**
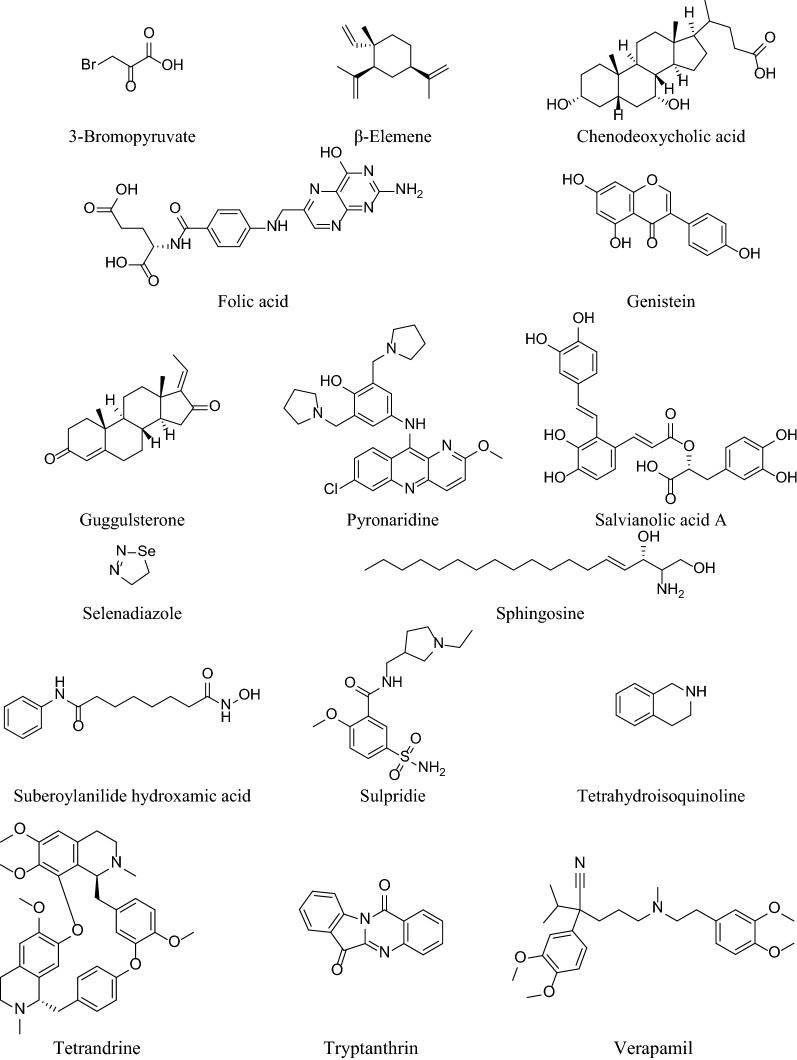
Chemical structure of some synthetic derivatives that acting against multi-drug resistant MCF-7 cell-line

Table 2 summarizes the synthetic derivatives acting against multi-drugresistant MCF-7 cell-line and Fig. 4 represents the chemical structures of these compounds.

**Table 2:** Synthetic derivatives acting against multi-drugresistant MCF-7 cell-line

Synthetic derivatives	Multi-drug resistant cancer cell-line	Inhibitory concentration (IC_50_)/ Lethal concentration (LC_50_)	Mechanism of action	References
Ceramide analogues: Pyridine-C4-ceramide Benzene-C4-ceramide, Adamantyl-ceramide, 5R-OH-3E-C8-ceramide	SKBr3 and MCF-7/Adr tumor cell	**Pyridine-C4-ceramide**, 24 h: 16.7 ± 3.8 µM (SKBr3), 13.4 ± 2.9 µM (MCF-7/Adr tumor cell) **Benzene-C4-ceramide**, 24 h: 18.6 ± 4.2 µM (SKBr3), 45.5 ± 6.5 µM (MCF-7/Adr tumor cell) **Adamantyl-ceramide**, 24 h: 10.9 ± 4 µM (SKBr3), 24.9 ± 0.3 µM (MCF-7/Adr tumor cell) **5R-OH-3E-C8-ceramide, 24 h**: 183 ± 5.5 µM (SKBr3), 21.2 ± 9.8 µM (MCF-7/Adr tumor cell)	Unknown selective toxicity. Ceramide analogues acting as neoplastic agent might be the reason for cancer cell destruction. Selective high proliferation rate for tumor cells, selectively inhibited cell cycle	[89]
Sphingosine Stereoisomers	MCF-7/ADR	50 µM	Sphingosine stereoisomers reduce basal phosphorylation of the P-gp ion in MCF-7/ADR cells, suggesting inhibition of PKC-mediated phosphorylation of P-gp	[85]
Selenoesters and Selenoanhydrides (1–11)	MCF-7	Above 100 µM	Exerted significant cytotoxic activity of ketone containing selenoesters against MCF-7 and KCR cell lines and the Se-compounds acting synergistically with doxorubicin on the KCR cell line	[90]
Suberoylanilide hydroxamic acid (SAHA)	MCF-7	5 µM	SAHA induced caspase-independent autophagic cell death rather than apoptotic cell death in TAMR/MCF-7 cells	[91]
*O*-(4-Ethoxyl-Butyl)-Berbamine (EBB)	MCF-7/ADR, MCF-7	**MCF-7/ADR:** DOX + EBB (1 mM): 8.34 ± 0.16 µM DOX + EBB (3 mM): 1.**9** ± **0**.86 µM DOX + EBB (6 mM): 1.03 ± 0.09 µM **MCF-7:** DOX + EBB (1 mM): 0.53 ± 0.06 µM DOX + EBB (3 mM): 0.48 ± 0.08 µM DOX + EBB (6 mM): 0.40 ± 0.07 µM	G2/M arrest and apoptosis of MCF-7/ADR cells, accompanied by downregulation of the proteins cdc2/p34 and cyclin B1 and increased the levels of calcium ions	[92]
Genistein	MCF-7/Adr	73.89 µM	Induced cell-cycle arrest and apoptosis. Genistein treatment strongly inhibited HER2/neu but not MDR-1 expression at both the mRNA and protein levels. Genistein acted synergistically with doxorubicin by increased intracellular accumulation of doxorubicin and suppressed HER2/neu expression	[87]
Pyronaridine	MCF-7/ADR	4.4 µM	Pyronaridine mediates its MDR reversal activity by direct inhibition of the MDR-mediated efflux process. Pyronaridine significantly raised the antitumor activity of doxorubicin when given intraperitoneally or orally without increasing the toxicity of doxorubicin	[93]
1,4-Dihydropyridines (DHPs) 3-pyridyl methyl carboxylate and alkyl carboxylate moieties at C3 and C5 positions and nitrophenyl or hetero aromatic rings at C4	MCF-7	4.12 ± 0.7 µM **(A2B5)** 15.60 ± 2.1 µM **(A2B2)** 16.42 ± 1.3 µM **(A1B2)** 26.45 ± 2.4 µM **(A3B1)** 21.47 ± 0.7 µM **(A4B1)**	Compounds bearing 3-nitrophenyl **(A2B2, A3B2)** and 4-nitrophenyl **(A3B1, A4B1)** moieties at C4 significantly inhibited rhodamine 123 efflux showing the mechanism of MDR reversal in P-gp transporter modulation. Lowered resistance of MES-SA/DX5 to doxorubicin also exerted the anti-tumor effect	[86]
Salvianolic acid A (SAA)	MCF-7	56.0 µM	Anti-tumor activity is due to the hypersensitivity of the resistant cell to the elevated ROS by SAA, SAA-triggered apoptosis due to increased caspase activity, disrupted mitochondrial membrane potential, downregulation of Bcl-2 expression, and upregulation of Bax expression in the resistant cells	[94]
Guggulsterone	Drug-resistant MCF-7	6.67 ± 0.67 µM **(MCF-7/DOX 10 µM)**	MDR-reversal effect of Guggulsterone might be a valuable adjunct to chemotherapy. Increased intracellular accumulation of doxorubicin by Guggulsterone expressed both MRP1 and P-gp	[84]
β-elemene	Doxorubicin-resistant MCF-7	11.70 ± 0.85 µM **(Doxorubucin + β-elemene 30 µM)**	Increased intracellular accumulation of Doxorubucin and Rh123 via inhibition of the P-gp transport function in Doxorubucin-resistant MCF-7 cells show the anti-tumor activity	[95]
Verapamil	Doxorubucin-resistant MCF-7	Not mentioned	Verapamil treatment results in a significant decrease in MDR1 mRNA levels. Increased intracellular accumulation of doxorubicin was seen after verapamil treatment in MCF-7/DOX cells	[96]
5-N formylardeemin, a new ardeemin derivative	Doxorubucin and Vincristine resistant MCF-7	**DOX + F-Ard (5 µM):** 20.808 ± 0.962 µM **VCR + F-Ard (5 µM):** 0.121 ± 0.007 µM	Reversed MDR activities through inhibiting MDR-1 expression by 5-N formylardeemin	[97]
A series of14β-hydroxy-10-deacetylbaccatinIII (14-OH-DAB) analogues: Paclitaxel, Docetaxel, IDN 5102, IDN 5106, IDN 5108, IDN 5109, IDN 5111, IDN 5127	MDA-MBA-231, MCF-7ADRr	**Paclitaxel:** 2.4 nM **(MDA-MBA-231)**, 2600 nM **(MCF-7ADRr)** **Docetaxel:** 0.8 nM **(MDA-MBA-231)**, 700 nM **(MCF-7ADRr)** **IDN 5102:** 1.8 nM **(MDA-MBA-231)**, 250 nM **(MCF-7ADRr)** **IDN 5106:** 2.2 nM **(MDA-MBA-231)**, 320 nM **(MCF-7ADRr)** **IDN 5108:** 10 nM **(MDA-MBA-231)**, 2500 nM **(MCF-7ADRr)** **IDN 5109:** 1.5 nM **(MDA-MBA-231)**, 85 nM **(MCF-7ADRr)** **IDN 5111:** 3.2 nM **(MDA-MBA-231)**, 180 nM **(MCF-7ADRr)** **IDN 5127:** 10 nM **(MDA-MBA-231)**, 640 nM **(MCF-7ADRr)**	Induce cell cycle block at G2/M in a concentration-dependent manner. G1/G2 ratio, measured as the amount of cell block correlates significantly (p < 0.001) with apoptosis, evaluated in the sub-G1 region. This incident suggests G2/M-blocked cells underwent apoptosis	[88]
Adba-27a	MCF-7/ADR	13.7 µM	Exhibited dose-dependent human topoisomerase IIα inhibitory activity and dose-dependent growth inhibitory activity in several drug-sensitive and multidrug-resistant cancer cell lines	[98]
Synthetis 1,4-dihydropyridine derivatives: 2a-h, 3a-e and 4a-e	MCF-7	0.03 µM (GI_50_)	-	[83]
Tetrandrine	MCF-7/Adr	0.79 ± 0.09 µM (2.5 µM of Tet)	Inhibited P-gp-mediated drug efflux. Modulate MDR by increased intracellular drug accumulation by inducing a decrease in the fluidity of thecell membrane	[99]
Sulpridie	MCF-7/Adr	–	Enhanced the response to dexamethasone by antagonizing the dopamine D2 receptor. Decreased level of MMP-2, increased E-cadherin level and, inhibited cell colony formation showed an anti-tumor effect	[100]
Peptide B1	MCF-7	21.9 µM	Exerted their anti-cancer activity by disrupting the cell membrane and entering into the cytoplasm, before acting on the mitochondria and stimulating the release of cytochrome C	[101]
Folic acid- hydroxypropyl-β-cyclodextrin – polyethylenimine/doxorubicin/ small interfering RNA (FA-HP-β-CD-PEI/DOX/ siRNA)	MCF-7	–	Downregulating the antiapoptotic protein BCL2, resulted in improving the therapeutic efficacy of the coadministered doxorubicin by tumor targeting and RNA interference	[102]
3-Bromopyruvate	MCF-7	12.5 and 25 µM	decrease in the intracellular level of ATP and HK-II bioactivity, inhibition of ATPase activity, and a slight decrease in P-gp expression in MCF-7/ADR cells	[103]
Tetrahydroisoquinoline [6,7-dimethoxy-1-(3,4-dimethoxy)benzyl-2-(N–n-octyl-N0-cyano)guanyl-1,2,3,4-tetrahydroisoquinoline]	MCF-7	10 µM	MDR reversal activity by directly modulating the function of P-gp or indirectly inhibition of P-gp transport function through decreasing membrane lipid fluidity	[104]
β-amino ester	MCF-7	7.89 µg/mL	Inhibit P-glycoprotein activity by lowering mitochondrial membrane potentials and ATP levels. The enhanced antitumor effect might be attributed to PHP-mediated lysosomal escape and drug efflux inhibition	[58]
Chenodeoxycholic acid	MCF-7	31 µM	Reduced HER2 expression and inhibited EGF mediated HER2 and p42/44 MAPK phosphorylation in these Tam-resistant breast cancer cells	[105]
MHY218	MCF-7	0.65 μM and 1.1 μM	MHY218 inhibited the proliferation of TAMR/MCF-7 cells and induced cell cycle arrest (G2/M phase) and caspase-independent autophagic cell death as well as apoptotic cell death, both in vitro and in vivo	[106]
Glutathione S-transferases (GST)	MCF-7	2.4–4.3 µM	GST π inhibitor was more potent at inhibiting total cytosolic GST catalytic activity in the MCF-7/ADR cell line	[107]
Tryptanthrin	MCF-7	0.14 to 11.13 µM	Downregulate GSTp gene, accompanied by less GST activity, to partly confer its MDR-reversing effect in doxorubicin-resistant cells	[108]
Selenadiazole	MCF-7	6.15 µM	Activated the AMPK signaling pathway and enhanced the cellular uptake of doxorubicin then the production of ROS, DNA damage, mitochondrial fragmentation, and apoptosis	[109]

*AMPK* AMP-activated protein kinase, *DOX* doxorubicin, *HK-II* hexokinase II, *GST* glutathione S-transferase, *MAPK* mitogen-activated protein kinase, *MDR* multi-drug resistance, *MMP-2* matrix metallopeptidase 2, *MRP1* multidrug resistance-associated protein 1, *P-gp* P-glycoprotein, *PHP* pH-sensitive poly(β-amino ester)s polymers, *PKC* protein kinase C, *ROS* reactive oxygen species

## Conclusion

The most common type of cancer is breast cancer for women worldwide, and approximately 25% of all female malignancies that have a high appearance in most of the developed countries. The second leading cause of death due to cancer among females in the world is breast cancer. The mortality rate of breast cancer is higher than the other types of cancer. Recent studies give evidence that the synthetic derivatives give effective action against breast cancer cell lines and also give action against multi drug resistant in MCF-7 cell lines. This review offers a very large amount of data on the mechanism of action of synthetic derivatives on multidrug resistance and could provide the basis for the discovery of new drugs against breast cancer. Multi drug resistance of cancer cells during chemotherapy it has been associated with a different type of mechanisms that are including enhanced efflux of drugs, genetic factors (gene mutations, amplifications, and epigenetic alterations), growth factors, increased DNA repair capacity, and also elevated metabolism of xenobiotics. For this reason, further studies required for the future purpose to know more about synthetic derivatives activity against breast cancer and multi drug resistance breast cancer cell lines.

## Data Availability

Not applicable.
